# Arctigenin protects mice from thioglycollate‐induced acute peritonitis

**DOI:** 10.1002/prp2.660

**Published:** 2020-09-22

**Authors:** Jingyi Zhao, Ying Chen, Lijun Dong, Xin Li, Ruijie Dong, Dongmei Zhou, Chengzhi Wang, Xiangdong Guo, Jieyou Zhang, Zhenyi Xue, Qing Xi, Lijuan Zhang, Guangze Yang, Yan Li, Rongxin Zhang

**Affiliations:** ^1^ Guangdong Province Key Laboratory for Biotechnology Drug Candidates Institute of Basic Medical Sciences and Department of Biotechnology School of Life Sciences and Biopharmaceutics Guangdong Pharmaceutical University Guangzhou China; ^2^ Department of Immunology Key Laboratory of Immune Microenvironment and Diseases of Educational Ministry of China Tianjin Medical University Tianjin China

**Keywords:** acute peritonitis, arctigenin, inflammation, macrophage, neutrophil

## Abstract

Acute peritonitis is an acute inflammatory response of the peritoneal cavity to physical injury and chemical stimulation. Timely resolution of this response is critical to prevent further damage to the body, which can eventually lead to more severe chronic inflammation. Arctigenin (ATG) is the main active ingredient of the Chinese medicine *Arctium lappa*. In recent years, there have been an increasing number of studies on the anti‐inflammatory effect of ATG, but there have been few studies on the effect of ATG on acute inflammation, especially in acute peritonitis, which has not been reported. In this study, a mouse model of experimental acute peritonitis induced by thioglycolate (TG) solution was used to study the protective anti‐inflammatory effect of ATG against acute peritonitis and the relevant mechanism. Our results showed that, after 12 hours of TG treatment, ATG significantly reduced inflammatory cell infiltration in mouse tissues and inhibited the secretion and expression of interleukin‐6 (IL‐6) and tumor necrosis factor alpha (TNF‐α) in mice. ATG significantly reduced the percentage of CD11b^+^Ly6G^+^ neutrophils and F4/80^+^ macrophages in the spleen and peritoneal exudate. In addition, ATG significantly inhibited the expression of the chemokines CCL3 and CCL4 and the adhesion molecule CD62L on the surface of CD11b‐positive monocytes. ATG was observed to inhibit the phosphorylation of p65 and p38 in LPS‐stimulated RAW264.7 cells. In conclusion, ATG can improve the symptoms of TG‐induced acute peritonitis through immune regulation. ATG can reduce the inflammatory response in TG‐induced acute peritonitis in mice.

**Abbreviations**
ATG: arctigenin
IL: interleukin
LPS: lipopolysaccharide
PD: peritoneal dialysis
TG: thioglycolate
TNF: tumor necrosis factor

## 1 INTRODUCTION

In general, when the body suffers from acute inflammation due to trauma, infection, postischemic injury, toxicity or autoimmune injury, different immune cells in the body begin to dynamically change and exert complex activities; that is, different immune cells perform different functions. Neutrophils are the main participants in the body's self‐defense system. They play an important role in the innate immune response and are also the first line of defense for the immune system. They are of great significance for regulating immune function and the inflammatory response. Paradoxically, neutrophils are also a major mediator of tissue damage in various human diseases.^1^, ^2^ Neutrophils have a circulating half‐life of approximately 6‐8 hours in the early stage of inflammation,^3^ after which they undergo spontaneous apoptosis. However, during acute inflammation, due to a variety of factors such as proinflammatory mediators, neutrophils do not undergo apoptosis over time, resulting in significantly prolongation of their life span and enhanced activation. Activated neutrophils lead to persistent inflammation and continuous tissue damage and can cause organ failure in critically ill patients.^4^ In the later stage of inflammation, macrophages play a key role. They are become activated after receiving relevant signals, actively accumulate at the inflammatory site, and clear pathogens or apoptotic neutrophils through phagocytosis. When macrophages fail to clear harmful substances through phagocytosis, macrophages are recruited in large quantities, and if left unchecked, these activated macrophages produce large amounts of free radicals and lyase, the accumulation of which causes tissue damage, thus triggering various inflammation‐related symptoms.^5^, ^6^ Therefore, macrophages must be regulated in a timely manner during the inflammatory process. During acute inflammation, various pro‐inflammatory cytokines (such as IL‐6 and TNF‐α) participate in leukocyte recruitment by upregulating the expression of cell adhesion molecules and chemokines. Excessive production of pro‐inflammatory cytokines has negative effects on various tissues and organs of the body to varying degrees, which may make the disease more complex and diverse.^7^

Peritonitis is a common complication in peritoneal dialysis (PD) patients with end‐stage renal disease, and although fewer than 5% of peritonitis episodes lead to death, peritonitis is the direct or primary cause of death of approximately 16% of PD patients.^8^, ^9^, ^10^, ^11^ Intraperitoneal injection of sterile TG solution has been widely used to induce acute peritonitis in mice.^12^, ^13^ TG solution‐induced peritonitis mimics various aspects of complex acute inflammation, involving rapid recruitment and activation of immune cells first followed by the release of excessive proinflammatory mediators, which aggravate inflammation.^14^ In addition, morphologic damage to the peritoneum in long‐term PD patients is associated with the formation of advanced glycation end products (AGEs) of peritoneal dialysates,^15^ a process that may lead to failure of PD. TG solution contains AGEs,^16^ so investigation of TG solution‐induced acute peritonitis is expected to provide new insights for the treatment of PD‐related peritonitis. Timely and appropriate management of peritonitis is critical to the success of PD.

*Arctium lappa*, as a traditional Chinese medicine, resists infection, alleviates sore throat and eliminates phlegm. Modern research has shown that the main active ingredient of burdock is arctigenin (ATG), a lignin‐like compound. ATG has many pharmacological activities and has been widely studied in recent years. Studies have shown that ATG can play a neuroprotective role by inhibiting the activation of microglia and reducing pro‐inflammatory cytokine levels.^17^ ATG can protect brain tissue from nerve damage through anti‐inflammatory and anti‐apoptotic effects and improve the prognosis of enhanced convection enhanced delivery (CED).^18^ Moreover, ATG can inhibit inflammatory cytokines by inhibiting the MAPK and NF‐κB pathways, thus mitigating the inflammatory response of the colon in mice. It also protects against LPS‐induced lung inflammation and oxidative damage by inhibiting the MAPK, HO‐1, and iNOS pathways.^19^, ^20^ In vitro studies have also shown that ATG can inhibit the production of NO and the secretion of inflammatory cytokines (TNF‐α and IL‐6) in LPS‐stimulated macrophages, thus playing an anti‐inflammatory role.^21^ In addition, there have also been some reports on the antioxidative properties of ATG. Studies in rats have shown that ATG can improve antioxidation in skeletal muscle and thus improve the swimming endurance of rats.^22^ Previously, we have demonstrated that ATG can alleviate mice experimental autoimmune encephalomyelitis (EAE) in mice by inhibiting Th17 cell differentiation and proliferation and protecting mice from ConA‐induced acute hepatitis.^23^, ^24^ In the present study, a mouse model of acute peritonitis was established by intraperitoneal injection of TG to explore the therapeutic and immunomodulatory effects of ATG against experimental acute peritonitis. The results showed that ATG plays an anti‐inflammatory role in TG‐induced acute peritonitis by inhibiting immune‐related mediators.

Activation of the NF‐κB pathway is a key marker of inflammation. NF‐κB is a multiple subunit nuclear transcription factor that rapidly activates the transcription of various cytokines and chemokines in response to various stimuli and plays a crucial role in inflammation. Its proper activation boosts the immune system's defenses. It is responsible for upregulating pro‐inflammatory cytokines and chemokines during inflammation and is a key regulator of many pro‐inflammatory cytokines (such as TNF‐α, IL‐1, and IL‐6) and chemokines.^25^ In addition, the mitogen‐activated protein kinase (MAPK) family mediates basic biological processes and cellular responses to external stress signals, and the MAPK pathway controls cytokine synthesis and release during inflammatory responses.^26^ Studies have shown that LPS can rapidly activate MAPK pathways, the NF‐κB pathway and other signaling pathways that are involved in the production and activation of inflammatory mediators and positively regulate the expression of many inflammation‐related genes.^27^ In this study, we stimulated RAW264.7 cells with LPS to observe the anti‐inflammatory mechanism of ATG.

## 2 MATERIALS AND METHODS

### 2.1 Cell culture and animals

Mouse mononuclear macrophage leukemia RAW264.7 cells were kindly provided by Professor Zhang Ning from the university. The cells were cultured in Dulbecco's modified Eagle medium (Gibco) supplemented with 10% FBS (Gibco), and 1% penicillin‐streptomycin (Gibco) in a 5% CO_2_ humidified incubator at 37°C. Lipopolysaccharide (LPS) (Sigma) and arctigenin (Shanghai Aladdin Science Technology Company, Shanghai) were dissolved in distilled water and dimethyl sulfoxide (Solarbio, Beijing), respectively.

The mice used in this experiment were female C57BL/6 mice aged 7‐8 weeks purchased from the Beijing Academy of Military Medical Science (China). All mice were kept in the Tianjin Laboratory Animal Center in a specific pathogen‐free (SPF) environment at a room temperature of 20°C‐25°C with free access to food and water. Before the experiment, the mice were randomly divided into two groups and allowed to adapt to the new environment for 1 week. All experiments were completed in accordance with the NIH Guide for the Care and Use of Laboratory Animals (Institute of Laboratory Animal Resources, 2011). All mouse experiments were performed in accordance with the rules and ethics for animal experiments of the Laboratory Animal Science Department, Tianjin Medical University, Tianjin, China.

### 2.2 Induction and treatment of acute peritonitis

Brewer thioglycollate (TG) medium (BD Biosciences) was dissolved in distilled water at a concentration of 3% (weight/volume) to prepare TG solution. The prepared solution was autoclaved and precipitated by standing at room temperature for at least 1 month before use.^16^ Mice were intraperitoneally injected with 2 mL of TG solution to induce acute peritonitis. For the treatment of acute peritonitis, arctigenin (ATG) was dissolved in DMSO in accordance with the manufacturer's instructions, diluted with PBS, and intraperitoneally injected into mice (10 μg/g). The mice were randomly divided into two groups; control mice were given the same dose of PBS and DMSO as mice in the ATG group. ATG (purity ≥ 98%) was obtained from Shanghai Aladdin Science Technology Company. The mice were weighed and examined daily, ATG treatment was given every day, and on the third day of treatment, TG solution was intraperitoneally injected to induce acute peritonitis.

### 2.3 H&E staining

After the female C57BL/6 mice (n = 5) were sacrificed by cervical dislocation, the liver and peritoneum were dissected and postfixed in 4% paraformaldehyde (weight/volume) for at least 48 hours. The tissues were dehydrated in gradient ethanol, embedded in paraffin, and sectioned (7 μm). The sections were dewaxed with xylene, stained with hematoxylin and eosin for 5‐20 minutes, and mounted with neutral gum. Stained sections from ATG‐treated mice and control mice were examined under a light microscope to detect inflammatory cell infiltration.

### 2.4 Flow cytometry

The peritoneal cavity of each mouse was washed twice with 5 mL of PBS to fully extract the peritoneal exudate, and a single cell suspension was still isolated with a cell filter. Single‐cell suspensions were isolated from the spleens and the peritoneal exudates of mice from the control group and ATG groups by grinding and filtering the tissues through a cell strainer. Some of these cells were stimulated for 4 hours with Cell Stimulation Cocktail (plus protein transport inhibitors) (500×) (eBioscience) in an incubator at 37℃ with 5% CO_2_. Then, the cells were fixed, permeabilized, and stained for intracellular cytokines with a PE‐conjugated anti‐mouse IL‐6 (Sungene Biotech, Tianjin; Cat# M100I5‐09C; clone MP5‐20F3) or PE‐conjugated anti‐mouse TNF‐α antibody (Sungene Biotech; Tianjin; Cat# M100T12‐09D; clone XT311). Other cell surface markers were assessed with a FITC anti‐mouse CD11b antibody (Sungene Biotech; Cat# M10117‐02E; clone M1/70), a PE‐conjugated anti‐mouse CD62L antibody (BD Biosciences; Cat# 553151; clone MEL‐14), a APC‐conjugated anti‐mouse F4/80 antibody (Sungene Biotech; Cat# M100F1‐11C; clone BM8) and an APC‐conjugated anti‐mouse Ly6G antibody (BioLegend; Cat# 108411; clone RB6‐8C5). Nonspecific expression was detected with a homotypic control. The average fluorescence intensity (MFI) was analyzed using FlowJo 7.6 software (BD Biosciences).

### 2.5 Quantitative real‐time PCR

Total RNA was isolated using TRIzol reagent (Invitrogen, USA) according to the manufacturer's instructions and converted to cDNA using random hexamers and M‐MLV reverse transcriptase (Invitrogen). GAPDH was used as an internal control. The mRNA levels of various cytokines were detected by real‐time quantitative RT‐PCR. Quantitative polymerase chain reaction (qPCR) primers were designed for genes including IL‐6, TNF‐α, CCL3, CCL4, and GAPDH. qPCR was performed was performed using the quick‐start universal SYBR Green Master mix in accordance with the manufacturer's instructions and was run on the Applied Biosystems^®^ 7500 Fast real‐time PCR system (Applied Biosystems). All reverse transcription reagents were purchased from Takara (Takara). The relative mRNA levels of various cytokines in each sample are shown as the 2^−ΔCt^ values.

### 2.6 ELISA assays for TNF‐α and IL‐6

Tumor necrosis factor (TNF‐α) and interleukin‐6 (IL‐6) levels in mouse serum were evaluated with commercial ELISA kits (Multi Sciences Biotech, Hangzhou) according to the manufacturer's instructions.

### 2.7 Western blot analysis

The MAPK and NF‐κB signaling pathways were evaluated in RAW264.7 cells by Western blotting. The cells were treated with different concentrations of ATG (10, 20 or 40 μmol/L) and incubated for 24 hours in an incubator containing 5% CO_2_ at 37℃. Cells were stimulated with LPS for 30 minutes and collected. The cells were lysed with RIPA buffer (Thermal, Shanghai) containing protease mixture (Sigma‐Aldrich) to extract protein, and the protein concentration was measured with a BCA protein detection kit (Thermal). The extracts were boiled in SDS‐PAGE loading buffer at 99°C for 5 minutes, separated on 10% SDS‐PAGE gels, and transferred to PVDF membrane (Roche) at 180 V. The membranes were incubated overnight at 4°C with NF‐κB p65 (1:1000 Cell Signal Technology, Danvers; Cat# 8242; RRID AB_10859369), phospho‐NF‐κB p65 (Cell Signal Technology, Cat# 3033; RRID AB_331284 1:1000), p38 MAPK(Cell Signal Technology, Cat# 8690; RRID AB_10999090 1:1000), phospho‐p38 MAPK (Cell Signal Technology, Cat# 4511; RRID AB_2139682 1:1000), and β‐actin (ABclonal, Wuhan; Cat# AC026; RRID AB_2768234 1:200,000) primary antibodies, which were diluted in TBST containing 5% skimmed milk. Then, the protein membranes were incubated with a secondary antibody conjugated to horseradish peroxidase (anti‐rabbit, Cell Signal Technology; Cat# 7074, RRID: AB_2099233 1:2000) at room temperature, and the Western blot bands were detected with ECL assay reagent (Millipore Corporation, Billerica).

### 2.8 Statistical analyses

The experimental analysis was carried out on three different occasions in triplicate. The data are expressed as the mean ± SD. Student's *t* test and ANOVA were used to perform statistical analyses. *P* < .05 indicated that the differences were statistically significant. The statistical graph was drawn using GraphPad Prism 8.

## 3 RESULTS

### 3.1 The protective effect of ATG against histologic injury in mice with TG‐induced acute peritonitis

**FIGURE 1:**
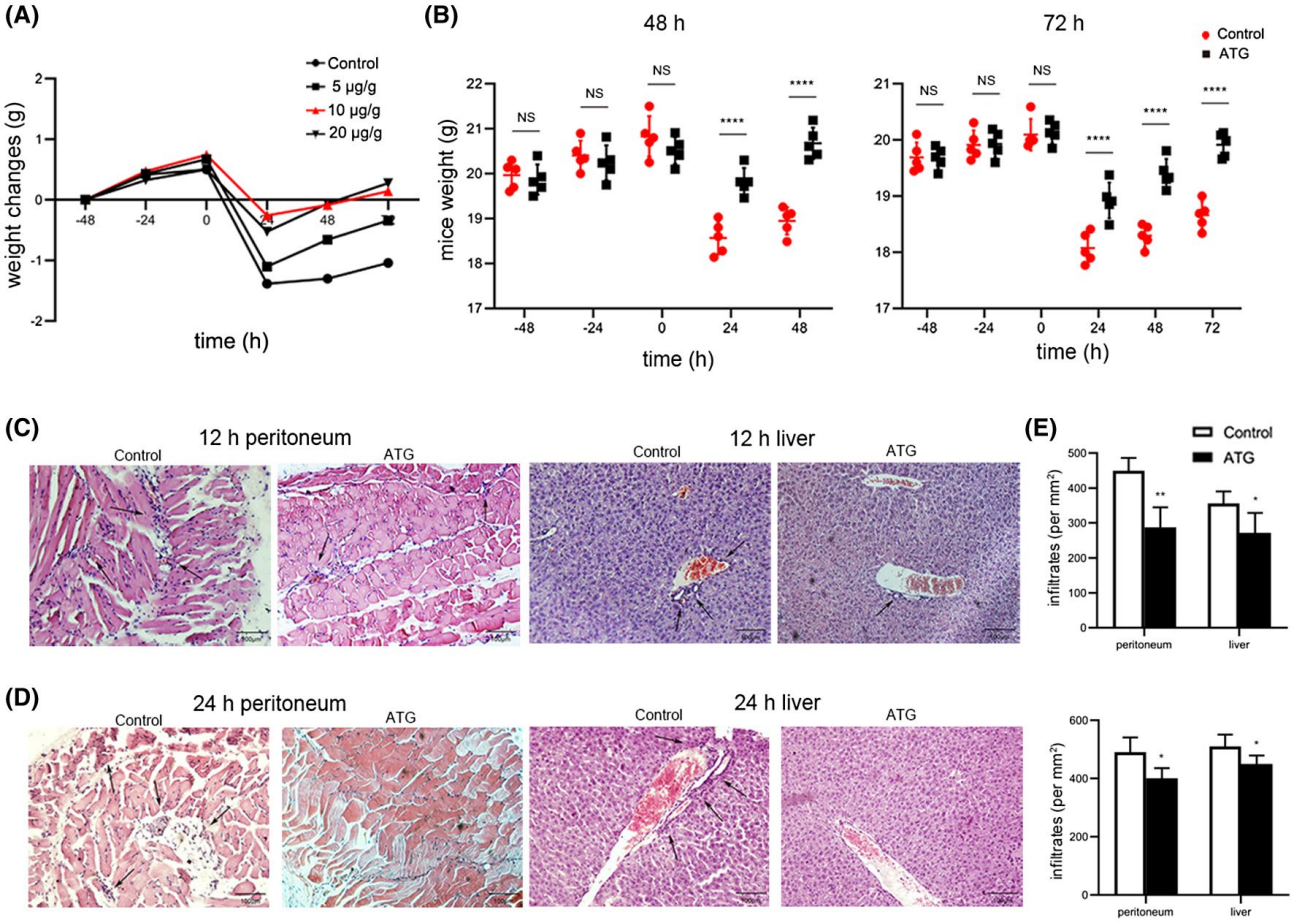
Effects of ATG on body weight and histological changes in mice. A, The line graph shows the effect of different doses of ATG (5 μg/g, 10 μg/g or 20 μg/g) on weight loss and recovery in mice with acute peritonitis. The doses of 10 μg/g and 20 μg/g have better effects on weight recovery of mice. In current study, we chose a dose of 10 μg/g (Red upright triangle) for research. B, The statistical graph shows the weights of the two groups of mice before and after acute peritonitis in the two groups, with 0 h representing the time of peritonitis induction (n = 5 mice per group). C, Twelve hours after TG injection, H&E staining of liver and peritoneal sections from mice in the control group and the ATG group showed infiltration of inflammatory cells in the tissues. D, Twelve hours after TG injection, H&E staining of peritoneal and liver sections from mice in the control group and the ATG group showed infiltration of inflammatory cells in the tissues. E, Quantitation of the infiltration in peritoneal and liver sections shown in C and D. The data represent at least three experiments with similar results. Representative images are shown. (scale bars: 100 µm; each bar represents the mean ± SD; 5 mice in each group; *P < .05, **P < .01, ****P < .0001; ANOVA and Student's *t* test)

Various diseases often have a significant impact on diet and weight. We treated mice with different doses of ATG (5 μg/g, 10 or 20 μg/g) and induced acute peritonitis, and observed the weight change of mice within 72 hours after TG treatment, as shown in Figure 1A (n = 5 mice per group). At the doses of 10 and 20 μg/g, weight of the mice decreased slowly compared with the control group and recovered relatively quickly. Therefore, in subsequent experiments, we chose a drug dose of 10 μg/g for following research.

To explore the effect of ATG on acute peritonitis, the weight of the two groups of mice were recorded every day during the period of ATG treatment and acute peritonitis induction by TG, and the weight changes of the mice at different time points were plotted in a statistical graph (Figure 1B). The mice were divided into the control group and ATG treatment group. It was found that the weights of the mice from both groups increased normally on the 2 days before TG injection, indicating that ATG had no significant influence on the weights of healthy mice. After the mice were intraperitoneally injected with TG, an acute local inflammatory response was induced in the peritoneal cavity. Within 24 hours after the injection, the weights of both groups of mice rapidly decreased significantly. In the 48‐hour experiment, 24 hours after TG induction, the weight of mice in the control group decreased by 2.26 g, while that of mice in the ATG group decreased by 0.232 g, with significant differences (each bar represents the mean ± SD; n = 5; ****P < .0001; ANOVA). Also in the 72‐hour experiment, 24 hours after TG induction, the weight of the two groups of mice decreased, the weight of the control group decreased by 2.018 g, and the weight of the experimental group decreased by 1.214 g (each bar represents the mean ± SD; n = 5; ****P < .0001; ANOVA). Forty‐eight hours and 72 hours after injection, the mice gradually regained weight, and the ATG treatment group regained weight faster than the Control group. In the 48‐hour experiment, 48 hours after TG induction, the weight of mice in the control and the ATG group increased by 0.376 and 0.852 g, respectively (each bar represents the mean ± SD; n = 5; ****P < .0001; ANOVA). In the 72‐hour experiment, 48 hours after TG induction, the weight of the control group increased by 0.218 g, while the weight of the experimental group mice increased by 0.416 g (each bar represents the mean ± SD; n = 5; ****P < .0001; ANOVA). 72 hours after TG induction, the weight of the control group increased by 0.366 g, while the weight of the experimental group mice increased by 0.516 g (each bar represents the mean ± SD; n = 5; ****P < .0001; ANOVA). We noted that rapid weight loss in mice occurred within 24 hours after TG injection, so we speculated that acute inflammation in the abdominal cavity of mice might also occur during this period. Furthermore, the weight of the ATG group mice recovered quickly, indicating that ATG played an active role in recovery from acute peritonitis in mice (Figure 1B). At the cellular level, acute inflammatory responses are characterized by significant changes in the number of immune cells in tissues over time. We examined changes in the number of inflammatory cells in the liver and peritoneum of mice at different time points by H&E staining. Based on the changes in the body weights of the mice, acute inflammation may have occurred before 24 hours after acute peritonitis induction. Therefore, liver and peritoneal tissues were collected at 12 hours and 24 hours to study inflammatory cell infiltration. As shown in Figure 1C,D (scale bars: 100 µm), there were many inflammatory cells around the blood vessels of liver tissues in the PBS treatment group. In the ATG treatment group, few inflammatory cells had infiltrated the liver tissues. However, a large number of inflammatory cells were observed in peritoneal tissues in the Control group. In the ATG treatment group, a small number of inflammatory cells were seen infiltrating the peritoneal tissue. Figure 1E shows the quantitative analyses of inflammatory cell infiltration in the peritoneum and liver of the two groups of mice at 12 and 24 hours after TG induction (each bar represents the mean ± SD; n = 5; *P < .05, **P < .01; Student's *t* test). These findings suggest that ATG can significantly inhibit the infiltration of inflammatory cells in tissues and improve the clinical symptoms of acute inflammation.

### 3.2 ATG suppresses neutrophils, macrophages, and chemokines in mice with acute peritonitis

**FIGURE 2:**
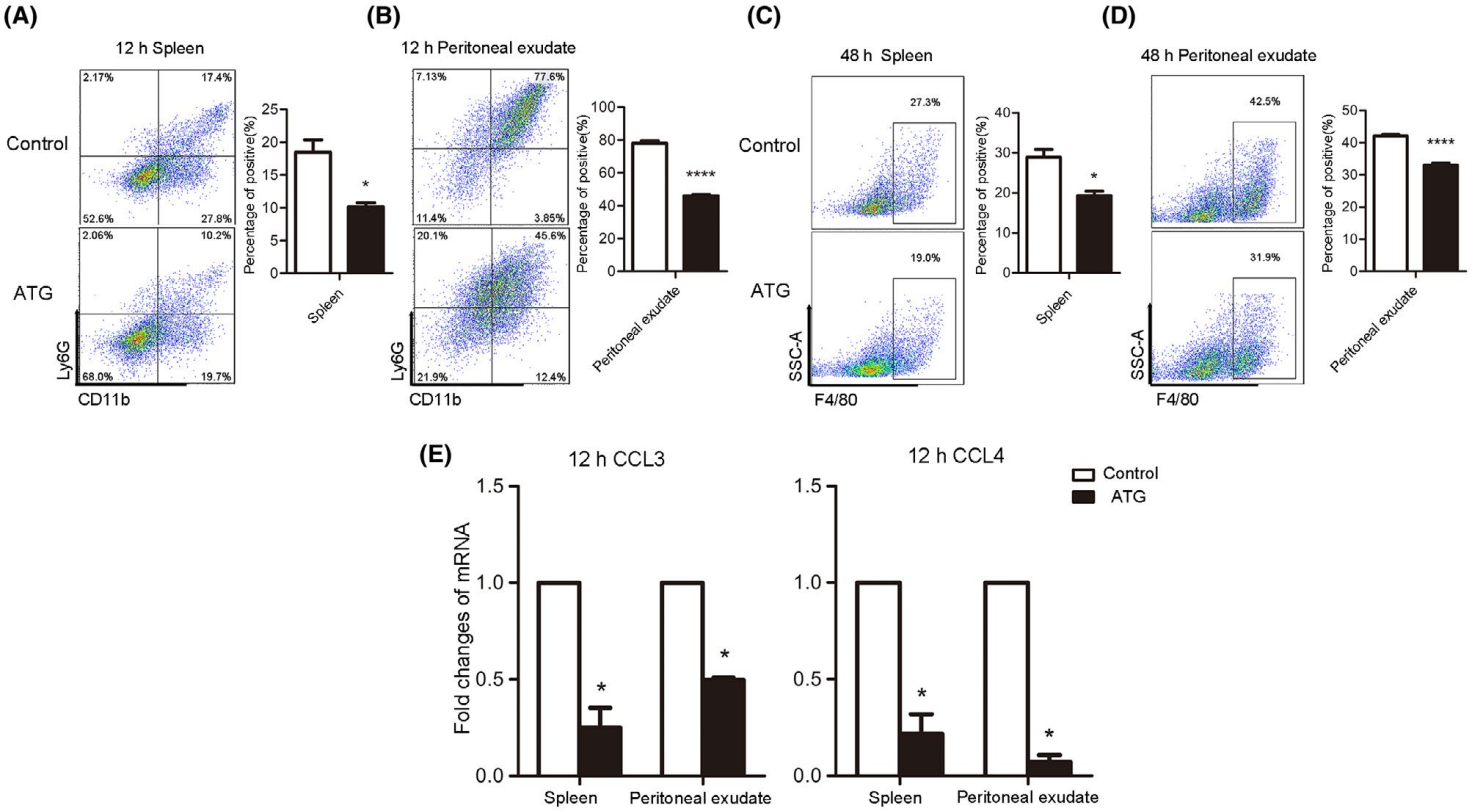
ATG suppresses neutrophils, macrophages, and chemokines in acute peritonitis mice. (A and B) Changes in neutrophils in the spleen and peritoneal exudate were detected by flow cytometry 12 h after TG injection. (C and D) Changes in F4/80 in the spleen and peritoneal exudate were detected by flow cytometry 48 h after TG injection. (E) Histogram showing statistical analysis of the mRNA expression of the chemokines CCL3 and CCL4 in the spleen and peritoneal exudate 12 h after TG injection. (each bar represents the mean ± SD; 5 mice in each group; *P < .05, ****P < .0001; Student's *t* test)

In the above experiments, we observed that inflammatory cells in the tissues of acute peritonitis mice were inhibited by ATG treatment, but the specific immune cells that are involved remains to be explored. Neutrophils and macrophages are a permanent topic of research related to the development of inflammation. The body's immune regulation of the ratio of neutrophils and macrophages plays an important role in the process of acute peritonitis and has a significant impact on the remission of inflammation and tissue healing. Therefore, we used flow cytometry to detect changes in the percentages of neutrophils and macrophages in mice. The results showed that ATG significantly reduced the percentage of neutrophils in the spleens and abdominal exudates of the mice 12 h after inflammation (Figure 2A,B) (each bar represents the mean ± SD; n = 5; *P < .05, ****P < .0001; Student's *t* test). Interestingly, a significant reduction in the percentage of macrophages occurred 48 h after TG injection (Figure 2C,D) (each bar represents the mean ± SD; n = 5; *P < .05, ****P < .0001; Student's *t* test). These findings may be consistent with the mechanism of immune cell regulation at the onset of acute peritonitis. At first, neutrophils mainly participate in combatting acute peritonitis, after which macrophages join the fight. Cellular chemotaxis is one of the important factors affecting the proportion of immune cells in the abdominal cavities of patients with acute peritonitis. Chemokines are classic cytokines that specifically regulate chemotaxis between cells. We further observed the mRNA expression levels of the chemokines CCL3 and CCL4 in the mouse spleen and peritoneal exudate 12 hours after peritonitis induction. As shown in Figure 2E, ATG significantly reduced the secretion of CCL3 and CCL4 (each bar represents the mean ± SD; n = 5; *P < .05; Student's *t* test). In summary, ATG reduces inflammatory damage by reducing the numbers of chemokines and neutrophils as well as macrophages.

### 3.3 ATG reduces TNF‐α and IL‐6 levels in acute peritonitis mice

**FIGURE 3:**
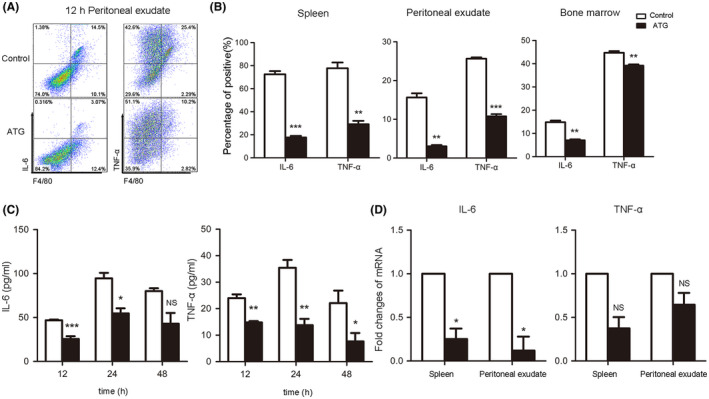
ATG reduces TNF‐α and IL‐6 levels in mice with acute peritonitis. A and B, Twelve hours after TG injection, flow cytometry was performed to analysis the spleens, peritoneal exudates and bone marrow cells of mice. IL‐6 and TNF‐α levels were significantly reduced after ATG treatment. A, The percentages of IL‐6 and TNF‐α produced in the experimental group and the control group are shown. B, The bar graph shows the statistical data analyzed by flow cytometry. C, Changes in serum IL‐6 and TNF‐α levels were detected by ELISA. The data in the bar graph were statistically analyzed. D, Changes in IL‐6 and TNF‐α levels in the spleen and peritoneal exudate were detected by RT‐PCR, and the data in the histogram were statistically analyzed (each bar represents the mean ± SD; 5 mice in each group; NS *P* > .05, *P < .05, **P < .01, ***P < .001; Student's *t* test)

During acute inflammation, the cytokines IL‐6 and TNF‐α are released in large quantities over a short period of time and are involved in the regulation of inflammation and the immune response. We speculated that ATG also has effects on proinflammatory cytokines; thus, we analyzed changes in the expression and secretion of IL‐6 and TNF‐α, the most representative pro‐inflammatory cytokines in acute inflammation, in the mouse serum at different time points. ELISA kits were used to detect serum levels in the two groups of mice 12, 24, and 48 hours after TG injection. The results showed the serum levels of the inflammatory cytokines IL‐6 and TNF‐α were significantly lower in the ATG group than in the control group (Figure 3C) (each bar represents the mean ± SD; n = 5; NS *P* > .05, *P < .05, **P < .01, ***P < .001; Student's *t* test). To further determine the effect of ATG on inflammatory factors, the mRNA expression levels of IL‐6 and TNF‐α in the mouse spleen and peritoneal exudate were measured. Twelve hours after TG was intraperitoneally injected into the mice, mice spleens and peritoneal exudate cells were collected to detect changes in the mRNA expression of genes of interest by RT‐PCR. It was found that ATG significantly reduced the mRNA expression of IL‐6 in the spleen and abdominal exudate. At the same time, the change of TNF‐α mRNA expression was also observed to decrease, but the difference was not significant (Figure 3D) (each bar represents the mean ± SD; n = 5; NS *P* > .05, *P < .05; Student's *t* test). Flow cytometry was used to detect the secretion of IL‐6 and TNF‐α in mouse tissues. The results showed that the secretion of IL‐6 and TNF‐α in the spleen, peritoneal fluid, and bone marrow was significantly reduced in the ATG group compared to the control group 12 hours after the onset of peritonitis (Figure 3A,B) (each bar represents the mean ± SD; n = 5; **P < .01, ***P < .001; Student's *t* test). In conclusion, ATG reduces the secretion of the proinflammatory cytokines IL‐6 and TNF‐α in acute peritonitis, thereby improving the symptoms of acute inflammation.

### 3.4 ATG reduces the expression of the neutrophil surface molecule CD62L

**FIGURE 4:**
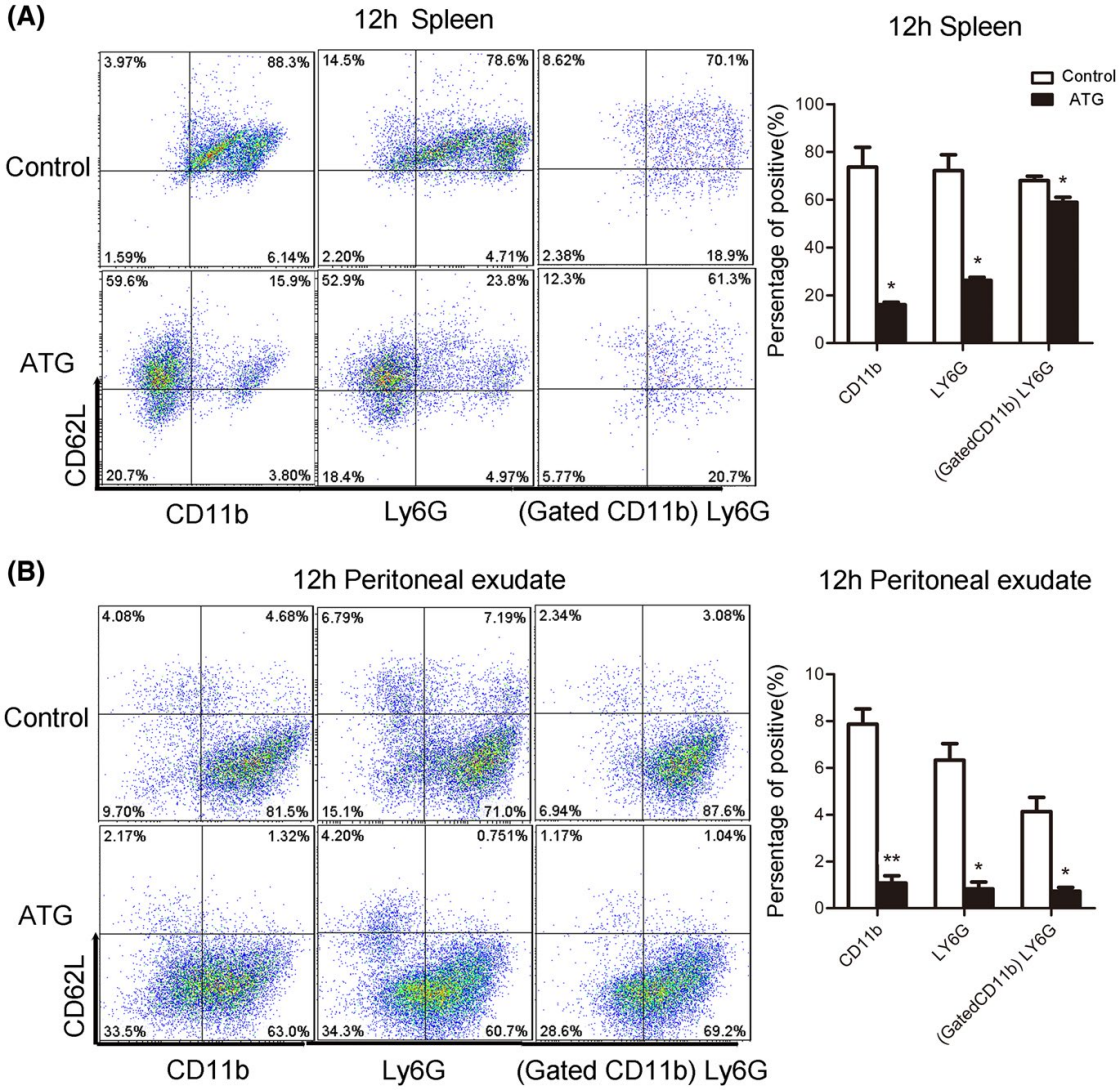
ATG reduces the expression of the neutrophil surface molecule CD62L. Twelve hours after TG injection, flow cytometry was performed to analysis the spleens and peritoneal exudates of mice. CD62L levels were significantly reduced after ATG treatment. A, Expression of CD62L in the spleen 12 h after TG injection. B, Expression of CD62L in the peritoneal exudate 12 h after TG injection (each bar represents the mean ± SD; 5 mice in each group; *P < .05, **P < .01; Student's *t* test)

CD62L is an adhesive receptor expressed by leukocytes and plays an important role in guiding leukocytes to migrate to the inflammatory site.^28^ The literature has shown that CD62L plays an important role especially in the initial infiltration of neutrophils in the inflammatory response.^29^ We measured changes in CD62L expression in both groups of mice. Flow cytometry was used to detect CD62L levels in the spleen and peritoneal exudate 12 hours after TG injection, and the results indicated that ATG significantly decreased the rate of chemotaxis of CD11b‐positive and Ly6G‐positive cells toward CD62L‐positive cells (Figure 4) (each bar represents the mean ± SD; n = 5; *P < .05, **P < .01; Student's *t* test). In other words, ATG inhibits the expression of CD62L on the surface of neutrophils, thus reducing the recruitment of neutrophils to the inflammatory site and reducing the inflammatory response, and this phenomenon could be one of the mechanisms underlying the anti‐inflammatory effect of ATG.

### 3.5 ATG regulates the NF‐κB and p38 MAPK pathways in RAW264.7 cells after treatment with LPS

**FIGURE 5:**
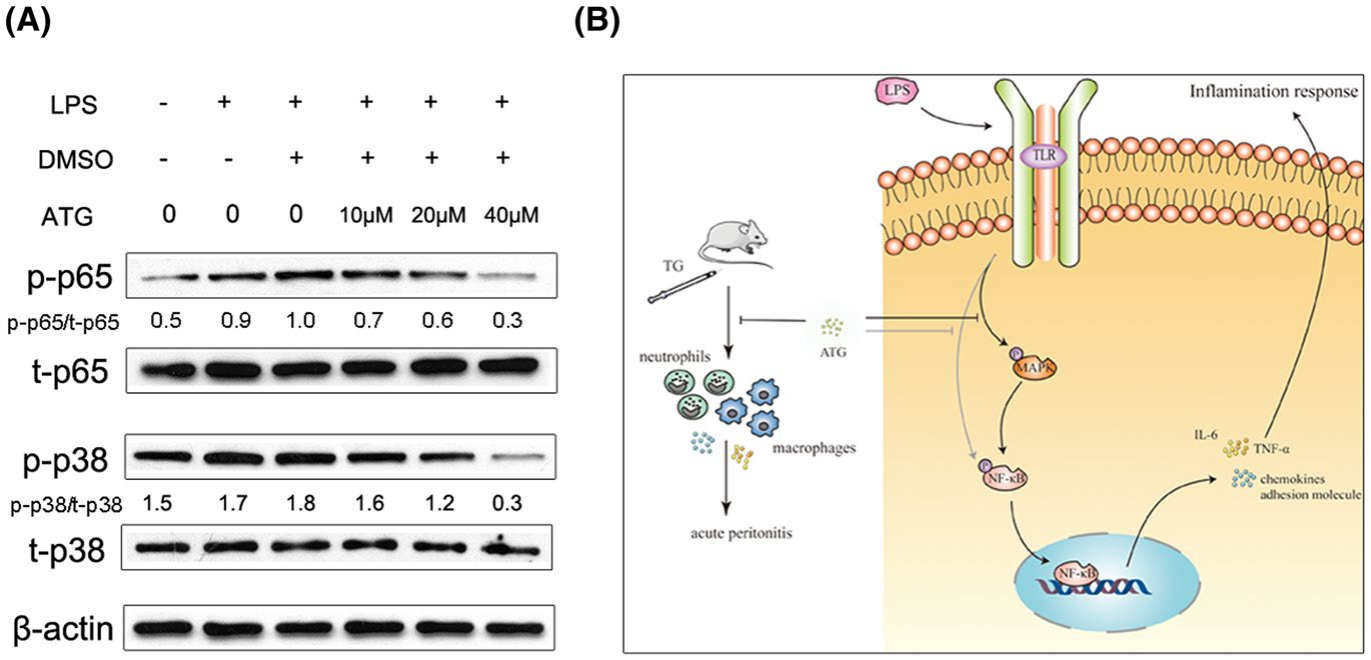
ATG regulates the NF‐κB and p38 MAPK pathways in RAW264.7 cells after treatment with LPS. A, RAW264.7 cells were treated with LPS (500 ng/mL) and different concentrations of ATG (10, 20, 40 μmol/L) for 24 h. There is a dose‐dependent decrease in the phosphorylation of p‐p65 and p‐p38 after treatment with ATG. B, Mechanism diagram for the effect of ATG on mice and cells

Next, the effects of ATG on the inflammatory pathways NF‐κB and MAPK pathways in LPS‐stimulated RAW264.7 cells were investigated by Western blotting. LPS is one of the most effective macrophage activators and can induce phosphorylation of p65 and p38 in RAW264.7 cells. We observed a dose‐dependent decrease in the expression of p‐p65 and p‐p38 after treatment with different concentrations of ATG (10, 20 or 40 μmol/L) (Figure 5A) (The Western Blotting used image J software to analyze the gray value, and used Graph Prism 8 to analyze the data). The possible mechanisms by which ATG regulates TG‐induced acute peritonitis in mice were shown in Figure 5B. The ATG inhibits the expression of proinflammatory mediators may by inhibiting the phosphorylation of p65 and p38 in RAW264.7 cells. In addition, ATG reduced the recruitment of neutrophils and macrophages in mice, and inhibited the expression of pro‐inflammatory mediators. These processes represent the mechanisms by which ATG relieves acute peritonitis in mice.

## 4 DISCUSSION

The inflammatory response can be divided into acute inflammation and chronic inflammation according to the onset time and pathological characteristics. Acute inflammation can occur when the body is stimulated or invaded by pathogens. After the onset of acute inflammation, under the action of chemokines and adhesion molecules, many immune cells are locally mobilized to fight the infection or pathogen; at the same time, immune cells release many cytokines to fight the infection. Large number of immune cells and infiltration and release of pro‐inflammatory cytokines by these immune cells can lead to excessive immunity, which can induce the development of acute inflammation into chronic inflammation and even cause secondary organ dysfunction or death.^30^, ^31^, ^32^ ATG is a natural lignan in burdock seeds that has pharmacological antiviral, antioxidant, anti‐inflammatory, and antitumor effects.^33^ In the past few years, our team has conducted relevant studies on the role of ATG in alleviating EAE in mice and protecting mice from conA‐induced acute liver injury. In the present study, we selected a dose of ATG with a significant therapeutic effect to investigate its effect on TG‐induced acute peritonitis in mice.^23^, ^24^ A major inflammatory response in the early stages of inflammation involves inflammatory mediators secreted by leukocytes, including the pro‐inflammatory cytokines IL‐6 and TNF‐α, which produce generate immune responses to target cells or tissues through specific receptors and signaling pathways.^34^ However, large concentrations of pro‐inflammatory cytokines can lead to inflammation and even necrosis in local tissues. Elevated levels of TNF‐α in the circulation have been reported in some inflammatory and infectious conditions, further confirming the pathogenic association between excessive production of TNF‐α and organ damage, and high expression of IL‐6 is found in some types of acute infection or inflammation.^35^ Our work showed that ATG can significantly reduce inflammatory cell infiltration and the secretion of IL‐6 and TNF‐α in the tissues of mice with acute peritonitis, thereby alleviating inflammation and histological damage.

After the mice were stimulated with TG, a large number of PMNs and macrophages are recruited to the inflammatory areas and tissues, causing an excessive immune response and thus triggering inflammation.^36^ During the early stage of acute peritonitis, PMNs and mononuclear cells accumulate (1‐4 hours),^37^ after which macrophages infiltrate the abdominal cavity (40‐72 hours), and the levels of proinflammatory cytokines in the peritoneal exudate and related tissues increase.^38^, ^39^, ^40^, ^41^ We observed that after ATG treatment, the numbers of neutrophils and macrophages in the peritoneal exudates and spleens of mice were significantly decreased at various time points and that the decreases in the number of these cells played a beneficial role in the timely control of inflammation.

When acute inflammation occurs, neutrophils and other immune cells need chemical attractants to help them migrate to the site of damage or infection sites, known as chemokines. Chemokines are large and diversified cytokines that can cause leukocytes to move toward the site of chemical stimulation and induce infiltration of various leukocytes when inflammation occurs in the body. They play a key role in physiological and pathological immune regulation. Functionally, chemokines fall into two broad categories. Some chemokines play a role in immune monitoring and act as homeostasis cytokines.^42^ Other chemokines are produced by cells only after infection or proinflammatory stimulation; these chemokines encourage white blood cells to migrate to the site of injury or infection. Our current study revealed that the levels of the chemokines CCL3 and CCL4 in the spleens and peritoneal exudates of mice with peritonitis were significantly reduced in the ATG group compared to the control group, suggesting that inflammation was alleviated, and this alleviation of inflammation also caused the significant reductions in the numbers of neutrophils and macrophages.

CD62L (L‐selectin) is an adhesion molecule expressed on neutrophils and monocytes and can be used clinically as a plasma/serum biomarker to stimulate leukocyte activity during inflammation. It has been reported that recruitment of polymorphonuclear neutrophils (PMNs) at inflammatory sites throughout the body is caused by CD62L mediated endothelial adhesion.^43^ Immediately after tissue damage, leukocytes in the circulation begin to interact with the vascular endothelium by rolling along the vascular wall. CD62L plays an important role in this process, directly mediating inflammation and leukocytes rolling at the site of tissue damage, and also exerts a significant impact on neutrophils.^28^ A significant decrease in neutrophil recruitment is observed in CD62L‐deficient mice in a 4‐hour experimental peritonitis model.^29^ Studies have shown that CD62L directly mediates rolling on endothelium at sites of inflammation or tissue injury in vivo.^44^ Our results showed that ATG can reduce the expression of CD62L, thus reducing its regulatory effects on leukocytes.

LPS induces inflammation by activating the NF‐κB and MAPK signaling pathways, and LPS stimulation increases the phosphorylation of p65 and p38 in RAW264.7 cells.^45^ It has been reported that activation of the NF‐κB p65 and MAPK p38 pathways can increase the synthesis and secretion of pro‐inflammatory mediators (TNF‐α and IL‐1β) and aggravate the inflammatory response.^46^ MAPKs play a key role in signaling pathways during inflammation and transduce various extracellular signals to the nucleus.^47^ In the resting state, p65 and its inhibitors are present in the cytoplasm. When cells are stimulated by LPS, many signaling pathways are activated, including MAPK pathways, which activate the NF‐κB pathway. Phosphorylation of p65 is increased and enters the nucleus to upregulate the transcription of proinflammatory cytokine genes, eventually triggering the pro‐inflammatory response.^48^, ^49^ Our in vitro data showed that ATG can inhibit the phosphorylation of p38 and p65, thereby blocking signal transduction in activated macrophages, and we believe that ATG exerts its anti‐inflammatory effect by inhibiting the phosphorylation of p65 and p38.

In vivo studies have shown that ATG can reduce the infiltration of tissue inflammatory cells, reduce the accumulation of neutrophils and macrophages, regulate the adhesion activity of endothelial cells by inhibiting the secretion of IL‐6, TNF‐α, and chemokines, and improve the symptoms of acute peritonitis. These anti‐inflammatory effects of ATG in vivo maybe achieved by downregulating the MAPK and NF‐κB pathways.

In conclusion, our findings confirm that ATG can prevent the further development of acute peritonitis through immune regulation within a short period of time, which provides new insight for the treatment of clinical acute peritonitis (peritoneal dialysis peritonitis in particular) and new potential for ATG as a plant‐derived therapeutic agent.

## DISCLOSURE

The authors have no financial conflicts of interest.

## AUTHOR CONTRIBUTIONS

Participated in research design: J. Zhao, Y. Li, and R. Zhang. Conducted experiments: J. Zhao, Y. Chen, L. Dong, R. Dong and D. Zhou. Performed data analysis: J. Zhao, Y. Li, X. Li and C.Wang. Contributed to the guidance of experiments: X. Guo, J. Zhang, Z.Xue, Q. Xi, L. Zhang and G. Yang. Wrote the manuscript: J. Zhao, and Y. Li. Edited and revised manuscript: Y. Li and R.Zhang.

## Data Availability

All data generated and analyzed in the study are available from the corresponding author upon reasonable request.
